# Spatial quantitation of antibiotics in bone tissue compartments by laser-capture microdissection coupled with UHPLC-tandem mass spectrometry

**DOI:** 10.1007/s00216-022-04257-3

**Published:** 2022-08-10

**Authors:** Firat Kaya, Matthew D. Zimmerman, Rosleine Antilus-Sainte, Martin Gengenbacher, Claire L. Carter, Véronique Dartois

**Affiliations:** 1 Center for Discovery and Innovation, Hackensack Meridian Health, 111 Ideation Way, Nutley, NJ 07110 USA; 2 Department of Medical Sciences, Hackensack Meridian School of Medicine, 123 Metro Blvd, Nutley, NJ USA; 3 Department of Pathology, Hackensack Meridian School of Medicine, 123 Metro Blvd, Nutley, NJ USA

**Keywords:** Antibiotics, Bone penetration, Spatial quantitation, Laser-capture microdissection, MALDI mass spectrometry imaging

## Abstract

Bones are the site of multiple diseases requiring chemotherapy, including cancer, arthritis, osteoporosis and infections. Yet limited methodologies are available to investigate the spatial distribution and quantitation of small molecule drugs in bone compartments, due to the difficulty of sectioning undecalcified bones and the interference of decalcification methods with spatially resolved drug quantitation. To measure drug concentrations in distinct anatomical bone regions, we have developed a workflow that enables spatial quantitation of thin undecalcified bone sections by laser-capture microdissection coupled to HPLC/tandem mass spectrometry, and spatial mapping on adjacent sections by mass spectrometry imaging. The adhesive film and staining methods were optimized to facilitate histology staining on the same sections used for mass spectrometry image acquisition, revealing drug accumulation in the underlying bone tissue architecture, for the first time. Absolute spatial concentrations of rifampicin, bedaquiline, doxycycline, vancomycin and several of their active metabolites are shown for both small rodent bones and larger rabbit bones that more closely resemble human bone density. Overlaid MALDI mass spectrometry images of drugs and histology staining enabled the generation of semi-quantitative data from regions of interest within anatomical bone compartments. These data correlated with absolute drug concentrations determined by HPLC–MS/MS in laser-capture microdissection samples. Collectively, these techniques enable semi- and fully quantitative drug distribution investigations within bone tissue compartments for the first time. Our workflow can be translated to image and quantify not only drugs but also biomarkers of disease to investigate drug penetration as well as mechanisms underlying bone disorders.

**Graphical abstract:**

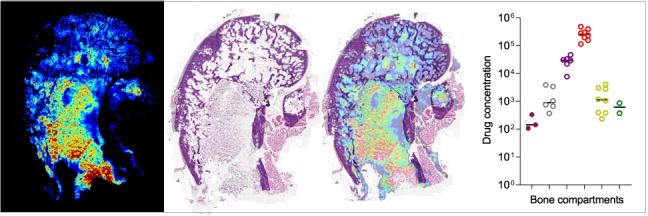

**Supplementary Information:**

The online version contains supplementary material available at 10.1007/s00216-022-04257-3.

## Introduction

Osteomyelitis requires prolonged antibiotic treatment, has a high recurrence rate, and can cause irreversible damage. With rising life expectancy, joint replacement is increasingly common, leading to increased opportunities for orthopedic device-related infections [1]. Therefore, treatment and surgical prophylaxis with antibiotics that reach the sites of infection at adequate concentrations are critical.

Early studies of antibiotic penetration into bone tissue measured drug concentrations using a bioassay such as the agar diffusion method [2], which was later replaced by high-performance liquid chromatography (HPLC) and liquid chromatography coupled to tandem mass spectrometry (LC–MS/MS), offering improved sensitivity and specificity [3]. Because bone is a heterogeneous and solid tissue, the major challenges associated with measurements of antibiotic concentrations in bones are sampling, processing, and drug extraction.

The organic and inorganic matrix provides strength and rigidity and constitutes > 90% of the bone tissue, with cells representing only 1–2% of total bone mass. The remaining tissue is made of blood vessels, nerves and lymphatics, organized in parallel and transversal canals. The thick and dense compact bone, or cortical bone, serves a mechanical function. Cancellous or spongy bone forms the inner part of the long bones, is relatively thin and porous, and serves physiologic functions. Cancellous bone has a higher degree of vascularization, a higher percentage of extravascular fluid, and a lower percentage of inorganic matrix than cortical bone, which can cause differences in antibiotic penetration [3, 4]. Importantly, different bacterial pathogens preferentially infect distinct bone compartments where they reside either extracellularly or within bone cells, but those sites and the mechanisms driving localized infections are not well known [5]. Thus, there is a critical unmet need to spatially quantify antibiotics in distinct cartilage and bone compartments susceptible to bacterial infection.

Classically, bone fragments frozen in liquid nitrogen are crushed with a mortar and a pestle, or pulverized under liquid nitrogen in a cryogenic mill, providing a very fine powder to optimize subsequent drug extraction. The latter method is compatible with thermally unstable drugs prone to degradation. Most published studies are based on such homogenized bone samples, and the total drug concentrations in bone homogenate are reported. Techniques to separate the different anatomical compartments of bone and measure concentrations in each are lacking [4]. Microdialysis has been used to determine unbound drug concentrations within interstitial bone fluid [6, 7], but it is not known whether the concentration measured represents the concentration that may occur at the site of infection [8]. 18F-Radiolabelled drug molecules have been used to determine concentrations of fluoroquinolones by positron emission tomography (PET) in bone from healthy volunteers. This method yields estimates of total, intra- and extracellular, average concentrations of drug per bone mass throughout the body [9, 10]. However, few antibiotics are amenable to [^18^F] PET tracer labelling that circumvent confounding imaging of metabolites in addition to the parent drug. While PET permits noninvasive measurement of drug concentrations over time in various tissues, including sites of infection, the approach is limited by its spatial resolution of approximately 1.0 cm^3^ and the short half-life of ^18^F. Mass spectrometry imaging of drugs in undecalcified human bone sections was recently reported. Through optimization of embedding media and supportive tapes, the authors demonstrated the detection and distribution of methadone and its metabolite, 2-ethylidene-1,5-dimethyl-3,3-diphenylpyrrolidine, in a section taken from postmortem human clavicle bone [11]. This elegant proof-of-concept study, while groundbreaking, does not provide absolute quantitative spatial analysis and due to the adhesive tape used, histological staining was not possible.

In recent years, our group has developed and optimized laser-capture microdissection (LCM) methods to quantify antibiotics at various sites of disease [12, 13] in soft tissue. Sectioning of decalcified bones provides high quality sections can be that preserve all essential microscopic elements, but the decalcification process is not compatible with spatially resolved drug quantitation due to antibiotic degradation and/or delocalization following treatment with 5–10% solutions of hydrochloric acid, nitric acid, or formic acid for up to 3 days. Though technically challenging, successful LCM of undecalcified bone sections is emerging for spatial transcriptomics profiling [14, 15]. Key to the success of this approach is the application of an adhesive film or tape on the cut surface of the sample to support the section and facilitate its transfer onto a slide or other support adequate for subsequent processing or imaging [16–20].

Here, we describe the sectioning, staining, imaging, laser-capture microdissection, and analytical methods we have developed to achieve spatial quantitation of rifampicin, doxycycline and vancomycin, broad-spectrum antibiotics, and bedaquiline, a new anti-tuberculosis drug also used in the treatment of nontuberculous mycobacterial infections, in undecalcified mouse and rabbit bones. We show that the methods are suitable to measure antibiotic concentrations in cortical bone, spongy bone and bone marrow of mice and rabbits, as well as in rabbit spinal cord and cartilage. We also describe a workflow for MALDI mass spectrometry imaging and histology staining of the same bone section. Our imaging workflow for the detection of bedaquiline in rabbit hip bone sections confirmed the quantitative data. This is the first report of spatially resolved quantitation of approved antibiotics in bone compartments that cannot be otherwise accurately separated for concentration measurements.

## Materials and methods

### Animal experiments

All animal studies were conducted in compliance with internationally recognized animal welfare guidelines and were approved by the Institutional Animal Care and Use Committee of Hackensack Meridian Health, Nutley, NJ. Four female CD-1 mice received a single 25 mg/kg oral dose of rifampicin formulated in 40% sucrose. In a second experiment, groups of 2 CD-1 mice (Charles River Laboratories) received a single 25 mg/kg oral dose of rifampicin formulated in water, 50 mg/kg subcutaneous dose of vancomycin in water, or 20 mg/kg oral dose of doxycycline in water. Mice were euthanized and femurs-tibia were harvested with the surrounding muscle tissue at 1 h (1st experiment) or 2 h (2nd experiment) post-dose. Three female New Zealand White (NZW) rabbits (Charles River Laboratories) weighing 2.2 to 2.6 kg were maintained under specific pathogen-free conditions and fed water and chow ad libitum. To rapidly achieve steady state, rabbits received three oral daily doses of bedaquiline at 160 mg/kg (loading dose) formulated in 20% hydroxypropyl β-D cyclodextrin adjusted to pH 3.0 with 50 mM citrate buffer. Rabbits were euthanized and femurs, tibia and vertebrae were collected 120 h after the third dose by pentobarbital overdose. Bone samples were flash-frozen in liquid nitrogen vapor and stored at − 80 °C until dissected. Serial blood samples were collected in K_2_-EDTA coated tubes between the last dose and the time of necropsy. Blood samples were processed to recover plasma and immediately stored at − 80 °C until analyzed for drug contents.

### Mimetic tissue preparation

Tissue mimetic were prepared as described [21]. Briefly, tissue homogenates were spiked with a small volume (< 2% v/v) of rifampicin, desacetyl-rifampicin, bedaquiline, desmethyl bedaquiline, doxycycline, and vancomycin standards of known concentrations. Spiked homogenates were frozen in cylindrical mold and frozen tissue plugs were mounted onto the cryostat and sectioned at 10 μm [21, 22]. Sections were mounted side-by-side on PET membrane slides with and without adhesive tape (Supplementary Fig. 1) and subjected to LCM and drug quantitation as described below.

### Bone tissue sectioning

Bone pieces were resized to a maximum of 3 × 2 cm using a band saw. Samples were embedded in 5% polyvinyl alcohol (PVA), snap frozen in liquid nitrogen, and stored at − 80 °C until sectioning. For cryosectioning, samples were mounted on the cryostat chuck using 5% PVA. Sectioning of undecalcified bone was performed with a 16 cm D profile tungsten carbide knife (DDK) using a Leica CM3050S (Leica) cryostat after mounting an adhesive cryofilm (cryofilm type 3C, 16UF, Section-Lab) onto the cut surface to support the Sect. [17]. The cryostat chamber temperature and object temperature were kept at − 30 °C and − 35 °C respectively. The clearance angle was set at 5 to 10°. Sections of up to 25 μm in thickness were collected onto the adhesive cryofilm to preserve the morphology of the tissue, and the quality of histology staining was compared across section thickness. For sections dedicated to LCM, the PET membrane was removed from the stainless-steel frame slides (Leica) and replaced with the adhesive cryofilm containing 10-μm-thick bone sections. These were then placed in airtight slide containers and stored at − 80 °C until dissection. For MALDI MSI, 5-μm-thick sections were sectioned using cryofilm and secured onto Superfrost Plus glass slides section facing up (Thermo Scientific) using double-sided adhesive film (3 M).

### Laser-capture microdissection

Laser-capture microdissection (LCM) was carried out using an LMD-7 laser microdissection system (Leica Microsystems) as described previously [13]. Samples were placed inside the slide holder of the microscope, tissue side facing down. Either 2.5 × or 5 × objectives were used to focus on the tissue. Laser parameters were 50, 120, and 12 for power, pulse, and offset respectively. Areas of approximately 6 × 10^6^ μm^2^ of cortical bone, cancellous (spongy) bone, cartilage, red marrow, and spinal cord compartments were collected in 0.2-mL PCR tubes (Corning 6571).

### Analytical methods for drug quantitation in plasma

Rifampicin, bedaquiline, vancomycin, doxycycline and verapamil were purchased from Sigma Aldrich; rifampicin-d8, bedaquiline-d6 and doxycycline-d5 were purchased from Toronto Research Chemicals. Drug-free K_2_-EDTA plasma from NZW rabbits and CD-1 mice was obtained from BioIVT for use as blank matrices to build standard curves. For the generation of calibration standards and quality control samples, stock solutions of rifampicin, bedaquiline, vancomycin and doxycycline were prepared in DMSO at a concentration of 1 mg/mL. Working solutions covering the desired concentration range for each drug were prepared by diluting the stock solutions in 50/50 acetonitrile (ACN)/Milli-Q water (MQW). Drug-free plasma (90 μL) was then spiked with 10 μL of the working solutions to create the calibration standards and QC samples. All standard and QC samples were prepared on the day of analysis. Drugs were extracted from 20 μL of the standard, QC and study samples by the addition of 180 μL of extract solvent containing internal standards (IS). The extraction solvents, internal standards, X-point calibration range, and QC sample information can be found in Supplementary Table 1. Five microliters of ascorbic acid was added to rifampicin samples to prevent spontaneous oxidation to the quinone [23]. Extracts were vortexed for 5 min and centrifuged at 5000 rpm for 5 min. One hundred microliters of supernatant for rifampicin and bedaquiline and 50 μL for vancomycin and doxycycline extracts were transferred to a 96-well plate for drug quantitation. LC–MS/MS analysis was performed on a SCIEX QTRAP 5500 triple-quadrupole mass spectrometer coupled to a SCIEX Exion UHPLC system. Multiple-reaction monitoring (MRM) of precursor/fragment transitions in electrospray positive-ionization mode was used to quantify the analytes. LC–MS/MS parameters are detailed in Supplementary Tables 2 and 3. Extracted ion chromatograms and MRM spectra for the four antibiotics are shown in Supplementary Fig. 2. Sample analysis was accepted if the concentrations of the quality control samples were within 20% of the nominal concentration. Data processing was performed using the Analyst software (version 1.7.2 Applied Biosystems Sciex).

### LCM sample processing for drug quantitation

Processing of LCM samples was performed as previously described [13]. Calibration and QC samples for bone quantitation were prepared by adding 10 μL of each working standard solution to 2 μL of 1:32.3 diluted in PBS drug-free rabbit bone dust. Drugs were extracted from the calibration standard, QC, blank/control, and LCM study samples by the addition of 50 μL of extract solvent containing IS (Supplementary Table 1). Extracts were sonicated for 10 min and centrifuged at 5,000 rpm for 5 min. Fifty microliters of supernatant was transferred to a 96-well plate for LC–MS/MS analysis, as described above.

### MALDI mass spectrometry imaging

Adhesive cryofilm containing 5-μm-thick sections were mounted on a Superfrost Plus microscopic glass slides (Thermo Scientific) using double-sided adhesive film (Cryofilm type 3C). Twenty-five milligrams per milliliter of 2,5-dihydroxybenzoic acid (DHB) in 2-propanol:tetrahydrofuran:MQW (45:45:10) with 0.1% trifluoroacetic acid (TFA) was applied to the tissue as matrix using an HTX M5 sprayer (HTX Technologies LLC). The following settings were used: 12 passes; 3 mm track spacing; 1200 mm/min velocity; 70 °C nozzle temperature; 8 psi nitrogen; 220 μL/min flow rate. Mass spectrometry imaging data were acquired using a Bruker SolariX 7 T FT-ICR mass spectrometer (Bruker Daltonics) equipped with a dual ESI/MALDI ion source and a smartbeam II Nd:YAG laser (355 nm). The instrument was operated in the positive ion mode using the CASI function, with the Q1 mass set at *m/z* 555 ± 200, covering the mass range of *m/z* 455–655. The small laser setting was used for all analyses, operated at a frequency of 2 kHz and 50 μm raster size. Data were imported as raw files into the SCiLS Lab MVS software, ver. 2020a Pro (Bruker Daltonics) for analysis. Regions of interest were drawn around the cortical bone, spongy bone, and red marrow. The spectral data from these regions were exported to GraphPad Prism (GraphPad Software, San Diego, CA, USA) for the generation of pixel intensity charts.

### Histology staining

Histological staining of sections on adhesive tape was adapted from the protocol published by Kawamoto and Kawamoto [17]. Briefly, bone tissue sections taken adjacent to the sections used for LCM were stained on the adhesive film with hematoxylin and eosin (aqueous). The sections were then mounted onto glass slides, with the section facing down (sandwiched between the slide and the cryofilm), using SCMM-G1 mounting medium (Section-Lab). This water-soluble media is used in place of xylene dehydration and convention mounting medium to avoid dissolving the tape material and adhesive. The slides were allowed to dry for 2 days prior to scanning. The stained slides were then scanned using a Pannoramic DESK II DW slide scanner (3DHISTECH). For post-MSI staining, the cryofilm tape was peeled off the glass slide, the matrix was washed off in running water, and the above protocol was repeated for on-tape H&E staining.

## Results and discussion

### Evaluation of tape interference during spatial drug quantitation

To assess possible interference of the adhesive film with drug extraction, ionization, and quantitation, tissue mimetic sections spiked with 10 μg/g of each drug were mounted either on steel frames with a PET membrane or on steel frames in which the PET membrane had been removed and replaced with the mimetic section on adhesive film (Fig. S1). Tissue mimetic sections were subjected to LCM followed by drug quantitation as described. Concentrations of the four study drugs and major active metabolites were within 20% of each other in the presence and absence of the adhesive tape, ruling out extraction and/or ionization effect caused by the tape and/or adhesive chemicals (Table 1).

**Table 1:** Impact of adhesive tape on drug quantitation

Drug/metabolite	Section on PET membrane		Section on adhesive tape		Diff (%)
	Raw analyte peak areas (× 1000)				
	Mean	SD	Mean	SD	
Rifampicin	26.85	54.45	32.20	3.82	− 18.1
Desacetyl-rifampicin	80.95	9.12	72.20	2.97	11.4
Bedaquiline	283.50	21.92	279.35	47.59	1.5
Desmethyl-bedaquiline	80.95	9.12	72.20	2.97	11.4
Doxycycline	20.65	2.19	20.45	0.78	1.0
Vancomycin	4.14	0.04	4.56	0.18	-9.7

### Sectioning of undecalcified bone for histology staining, laser-capture microdissection and MALDI MSI

**Fig. 1:**
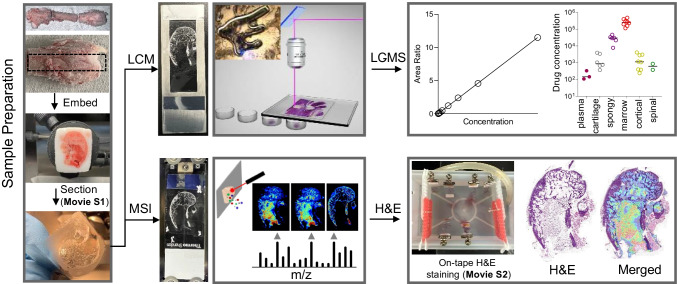
Workflow schematic from sample preparation to (**i**) spatial quantitation by laser-capture microdissection (LCM) coupled to UHPLC/tandem mass spectrometry (LC–MS) or (**ii**) MALDI mass spectrometry imaging (MSI) followed by same-section histology staining with hematoxylin and eosin (H&E)

**Fig. 2:**
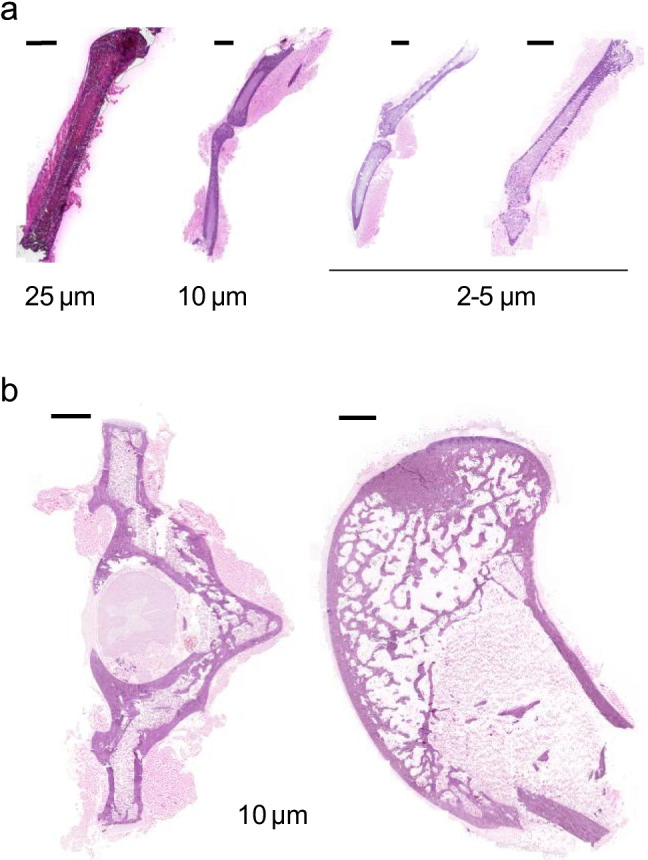
Histology staining of mouse and rabbit undecalcified bone sections. (**a**) Optimization of tissue section thickness for high-quality hematoxylin and eosin (H&E) staining of mouse bones (tibia and femur). (**b**) H&E staining of 10-μm-thick sections of rabbit vertebrae (left) and femur head (right). Scale bars: 2 mm

Sectioning of undecalcified and unfixed bone, while challenging, is required for histology staining, spatial drug quantitation and drug imaging, since both decalcification and fixation processes cause degradation and/or drug delocalization. Sectioning rodent bones is technically simpler than larger mammalian or human bone as it is less dense. A MALDI MSI method was first published in 2012 to image endogenous molecules [19] and has been adapted and further optimized since [24]. Two methods for the sectioning and MSI of undecalcified human bone were recently reported and were directed at lipid analysis in fixed tissues [20] and drug analysis in unfixed tissues [11]. We therefore developed a workflow based on these two previous studies to optimize a protocol compatible with LCM coupled to LC–MS/MS for drug quantitation and MALDI MSI followed by histological staining of the same section for both rodent and larger mammalian bone. A schematic of our workflow is shown in Fig. 1. The femur, femur head, knee, tibia and vertebrae were selected since they are common sites of infection due to joint replacement, injuries, and spinal tuberculosis. Carboxymethyl cellulose 3 or 5%, gelatin 15% and polyvinyl alcohol (PVA) 5% were explored as embedding medium. PVA was selected because it provided optimal sections that readily maintained bone integrity. Section thickness, sectioning speed (20% max) and clearance angle (10°) were optimized as needed to maintain integrity of the bone tissue. To determine the optimal section thickness for best quality of H&E staining, we stained side-by-side whole leg mouse sections of different thicknesses (Fig. 2). At 5- to 10-μm thickness or single cell layer, the best compromise between section integrity and staining quality was achieved.

### Quantitation of antibiotics in bone compartments by laser-capture microdissection and LC–MS/MS

Laser parameters were adjusted to account for the hardness and density of the bone tissue and optimized for each objective. A power setting of 50 was adequate to ensure complete cut while minimizing heating around the dissected area. This is important to prevent bone fragmentation, loss of bone mass, and drug degradation. Dissected areas were pooled until approximately 6 × 10^6^ μm^2^ were collected per tube, to ensure that the extracted drugs remained above the limit of quantitation. An extraction time course up to 24 h with bedaquiline (the most highly bound and hydrophobic study drug) was performed to identify the shortest sonication time required to effectively extract study drugs from 10-μm-thick section areas. We found that sonication for 1 to 5 min achieved optimal extraction after which the fraction of extracted drug no longer increased, in cortical bone spongy bone and red marrow (Supplementary Fig. 3). All study samples were sonicated for 10 min based on these findings.

**Fig. 3:**
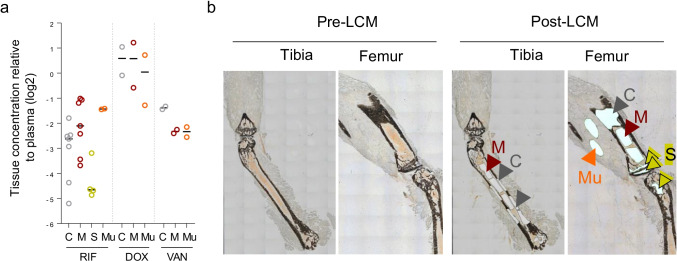
Determination of broad-spectrum antibiotic concentrations in mouse bone compartments by laser-capture microdissection (LCM) coupled to LC–MS/MS. (**a**) Concentration ratios of rifampicin (RIF, *n* = 6 bones from 5 mice), doxycycline (DOX, *N* = 2 bones from 2 mice) and vancomycin (VAN, *n* = 2 bones from 2 mice) in cortical bone (C), red marrow (M), spongy bone (S) and surrounding muscle tissue (Mu) relative to plasma concentrations obtained at the time of necropsy. (**b**) Schematic showing mouse bone sections pre- and post-LCM and dissected areas for each compartment shown in (a), using matching color coding

Next, we sampled tibia and femur bones from mice that had received single doses of rifampicin, doxycycline, or vancomycin, sectioned as described, isolated bone compartments and surrounding muscle tissue by LCM, and quantified each antibiotic in cortical and spongy bone, red marrow, and muscle tissue, relative to plasma (Fig. 3). Rifampicin concentrations were lower in all compartments than in plasma and variable across compartments. Vancomycin penetration was consistent across compartments with tissue-to-plasma ratios around 0.2 to 0.3. Of the three antibiotics, doxycycline penetrated bone tissue most effectively.

**Fig. 4:**
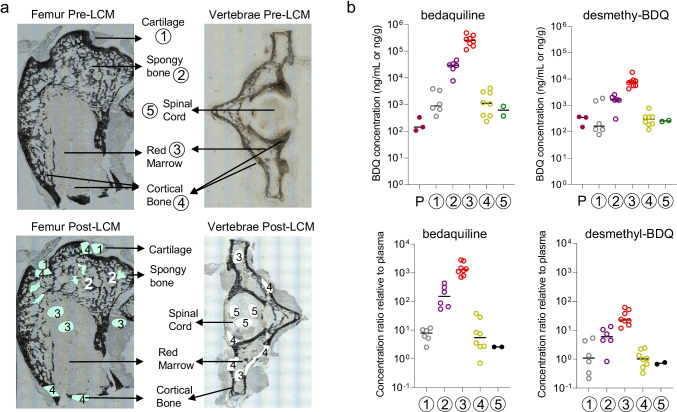
Quantitation of anti-tuberculosis agent bedaquiline and its major active metabolite in rabbit bone compartments by LCM coupled to LC–MS/MS. (**a**) Schematic showing rabbit femur head and vertebrae sections pre- and post-LCM, including the 5 anatomically distinct bone areas sampled by LCM. (**b**) Absolute concentrations (top row) and concentration ratios relative to plasma (bottom row) of bedaquiline and desmethyl bedaquiline (BDQ) in cartilage, spongy bone, red marrow, cortical bone and spinal cord. Compartment numbering as described in (a)

To demonstrate the applicability of our method to bones from larger animals more closely resembling human bones, and to allow for a longer period of drug equilibration from plasma to bone tissue, we administered 3 daily doses of bedaquiline to New Zealand White rabbits. Bedaquiline was chosen since it is known to exhibit differential partitioning in plasma, cellular and acellular tissue compartments [25] due to its unique physicochemical properties (cationic amphiphilic drug with cLogP > 7 and pKa ~ 13). Femur heads and vertebrae were collected and processed for LCM as described. Bedaquiline concentrations were highest in red marrow followed by spongy bone, 2 to 3 orders of magnitude higher than in plasma. In cartilage, cortical bone and spinal cord, bedaquiline concentrations were lower and similar. Desmethyl-bedaquiline concentrations followed the same trend but were consistently lower than those of the parent drug (Fig. 4). Importantly, the results were very consistent across samples and across animals, more so than in mice, possibly due to larger bones allowing for more accurate dissection of different compartments, as well as the narrow peak to trough window of bedaquiline throughout a dosing interval in rabbits at steady state (Supplementary Fig. 4). The highest drug penetration was observed in bone marrow, which could be partly explained by the superior and homogeneous vascular density compared to other bone compartments.

### Same section MALDI MSI of bedaquiline and histology staining

**Fig. 5:**
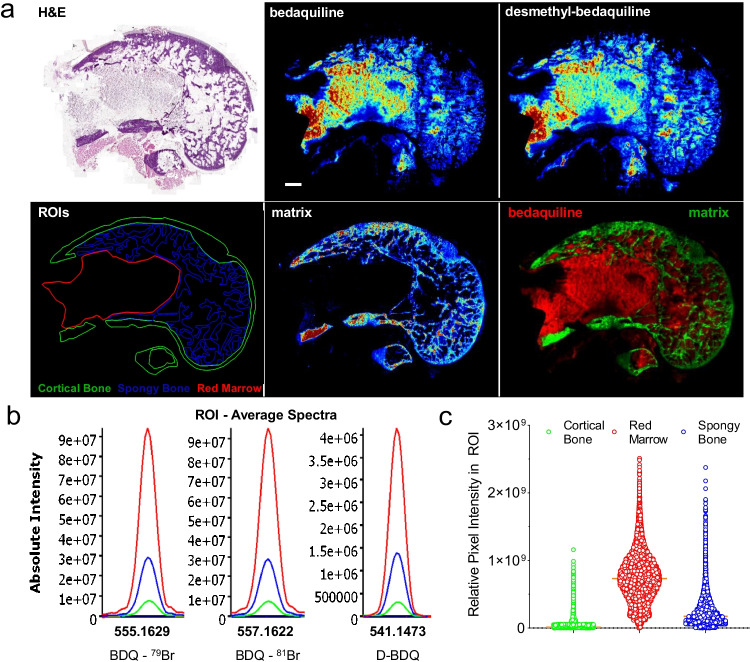
Semi-quantitative imaging of bedaquiline in rabbit femur compartments by MALDI mass spectrometry. (**a**) Top row: H&E-stained section and corresponding MALDI MS images of bedaquiline and its active metabolite (desmethyl bedaquiline). Lower left: delineation of the three major regions of interest based on H&E staining and MS images: cortical bone, spongy bone and red marrow; MS image of a matrix cluster ion at *m/z* 656.0529; and a merged ion image of bedaquiline and the matrix ion. (**b**) Average ion spectra of bedaquiline and desmethyl bedaquiline in the three regions of interest outlined in (a), showing the characteristic signature of the two naturally occurring bromine isotopes (**c**) Scatter plots of relative pixel intensities within the three regions of interest outlined in (a)

To correlate spatial quantitation with histologically defined regions and to confirm the spatial quantitation data obtained by LCM in rabbit bones, we carried out MS imaging of bedaquiline in femur sections directly adjacent to those used for LCM spatial quantitation (Fig. 1). Our workflow was developed to enable H&E staining on the same section used for MSI (Fig. 5a, top left). This will be of vital importance when carrying out investigations directed at drug penetration into infected bone tissue as this can help inform on therapeutic efficacy based on the local microenvironment. Bedaquiline and its major metabolite, desmethyl bedaquiline, were detected with highest abundance in the red marrow, followed by the spongy bone, with low signal detected in a few regions of cortical bone (Fig. 5a, top middle and right panel). Matrix ions are known to cluster and form adducts with components of cortical bone, such as fragment ions of hydroxyapatite, when DHB is used as the matrix [20]. As the imaging data were acquired over a small mass window, no intact endogenous lipids were detected that could be mapped to the spongy and cortical bone regions. Thus, to highlight the spongy and cortical bone regions, we used a matrix cluster ion and merged this ion image with bedaquiline to further demonstrate the variation in drug signal across these regions (Fig. 5a, bottom panel). The H&E and MS images were then merged and used to draw regions of interest (ROIs) for cortical bone, spongy bone and bone marrow (Fig. 5a, bottom left). The average ion spectra of bedaquiline and desmethyl bedaquiline in the three regions of interest, and the characteristic signature of the naturally occurring bromine isotopes (^79^Br and ^81^Br) in bedaquiline and its metabolite (Supplementary Fig. 5), are shown in Fig. 5b. To generate semi-quantitative data from these ion maps, the individual pixel intensities from the ROIs were plotted (Fig. 5c), confirming the partitioning of bedaquiline visualized by MSI and consistent with the spatial quantitation of bedaquiline by LCM (Fig. 4).

In summary, we have developed techniques and a workflow to spatially quantify and image the distribution of antibiotics in anatomically distinct bone compartments. Sectioning of undecalcified mouse and rabbit bones was optimized to enable absolute drug quantitation by LCM coupled to LC–MS/MS and MALDI MS imaging of a directly adjacent section. Adapting a more aqueous staining method compatible with adhesive film enabled on-tape histology staining post-MALDI image acquisition, a feature that will be critical when applying the methodology to diseased bone tissues where histopathologic abnormalities can impact drug penetration and efficacy.

Concordant results were obtained by LCM coupled to LC–MS/MS, ion maps acquired by MALDI MS imaging and average pixel intensities measured in major bone compartments. Optimal antibiotic penetration was seen in bone marrow, likely due to a dense and homogeneous vascular network.

This platform can now be applied to visualize and quantify antibiotics administered to treat a variety of bone infections, as well as drugs and drug candidates used against other bone pathologies, which will enable dose optimization. Our developed workflow is not limited to small molecule drugs. Animal models of bone disorders and human bones removed prior to joint replacement can be leveraged to image and quantify biomarkers of disease, such as microbial surface constituents, inflammatory signaling lipids and other molecules, to understand the mechanism underlying infection, rheumatoid arthritis, metastasis, bone degeneration, and other bone disorders.

## Supplementary Information

Below is the link to the electronic supplementary material.

Supplementary file1 (DOCX 6800 KB)

Supplementary file1 (MP4 35422 KB)

Supplementary file2 (MP4 5146 KB)
