# Supplementary material for: Spatial quantitation of antibiotics in bone tissue compartments by laser-capture microdissection coupled with UHPLC-tandem mass spectrometry
Source: Anal Bioanal Chem. 2022 Aug 10;414(23):6919–27. doi: 10.1007/s00216-022-04257-3 (PMC9436889; doi:10.1007/s00216-022-04257-3)
Supplement: Supplementary file 1 — Supplementary file1 (DOCX 6800 KB) [file 216_2022_4257_MOESM1_ESM.docx]

Spatial quantitation of antibiotics in bone tissue compartments by laser-capture microdissection coupled with UHPLC-tandem mass spectrometry

Firat Kaya ^(1)^, Matthew D. Zimmerman ^(1)^, Rosleine Antilus-Sainte ^(1)^, Martin Gengenbacher ^(1,2)^, Claire L. Carter ^(1,3) *^, and Véronique Dartois ^(1,2) *^

^(1)^ Center for Discovery and Innovation, Hackensack Meridian Health, 111 Ideation Way, Nutley, NJ

^(2)^ Hackensack Meridian School of Medicine, Department of Medical Sciences, 123 Metro Blvd, Nutley, NJ.

^(3)^ Hackensack Meridian School of Medicine, Department of Pathology, 123 Metro Blvd, Nutley, NJ.

^(*)^ corresponding authors

SUPPORTING INFORMATION

**Supplementary Table 1.** Sample Preparation Information

| **Drug Compound** | **Internal Standard** | **Extraction Solvent** | **Calibration Standards** | **Quality Control** |
| --- | --- | --- | --- | --- |
| Rifampicin (RIF) | rifampicin-d8 (5 ng/mL) | 50/50 acetonitrile/methanol | 10-50K ng/mL | 10, 100, 1K, 10K ng/mL |
| Bedaquiline (BDQ) | bedaquiline-d6 (5 ng/mL) | 50/50 acetonitrile/methanol | 10-500K ng/mL | 10, 100, 1K, 10K ng/mL |
| Vancomycin (VAN) | verapamil (500 ng/mL) | 33% trichloroacetic acid | 500-50K ng/mL | 1K, 10K ng/mL |
| Doxycycline (DOX) | doxycycline-d5 (250 ng/mL) | 33% trichloroacetic acid | 100-50K ng/mL | 100, 1K, 10K ng/mL |

**Supplementary Table 2.** Chromatography parameters

| **Antibiotic** | **Column** | **Column Temperature (C)** | **Injection Volume (µL)** | **Flow Rate (mL/min)** | **Mobile Phase A** | **Mobile Phase B** | **Rt (min)** |
| --- | --- | --- | --- | --- | --- | --- | --- |
| Rifampicin | Agilent Zorbax SB-C8 column (2.1x30 mm; particle size, 3.5 µm) | 30 | 2 | 0.6 | 0.1% FA in water | 0.1% FA in acetonitrile | 1.63 |
| Bedaquiline | Agilent Zorbax SB-C8 column (2.1x30 mm; particle size, 3.5 µm) | 30 | 1 | 0.6 | 0.1% FA in water | 0.1% FA in acetonitrile | 1.86 |
| Vancomycin | Agilent Eclipse XDB-C18 column (3.0 x 75 mm; particle size, 3.5 µm) | 30 | 10 | 0.6 | 0.1% FA in water | 0.1% FA in acetonitrile | 1.51 |
| Doxycycline | Agilent Zorbax SB-C8 column (2.1x30 mm; particle size, 3.5 µm) | 30 | 5 | 0.6 | *0.1% FA in water | *0.1% FA in acetonitrile | 1.03 |

^(*)^ 0.1 % heptafluorobutyric acid was added. FA = formic acid; Rt = retention time, MRM = multiple reaction monitoring; CE = Collision energy.

A 3.5-minute reversed-phase gradient method was used for all compounds starting with 5 % mobile phase B, ramped to 95% over 2.5 minutes, held and equilibrated until 3.5 minutes and then returned to 5 %.

**Supplementary Table 3.** Mass spectrometry parameters

| **Analyte** | **CUR (psi)** | **CAD** | **IS (V)** | **Temperature (℃)** | **GS1 (psi)** | **GS2 (psi)** | **DP (V)** | **CE (V)** | **CXP (V)** | **Ion** | **MRM transitions** |
| --- | --- | --- | --- | --- | --- | --- | --- | --- | --- | --- | --- |
| Rifampicin | 20 | Medium | 5500 | 450 | 55 | 60 | 101 | 25 | 36 | [M+H]^+^ | 831.50/799.60 |
| Bedaquiline | 30 | Medium | 5500 | 600 | 55 | 60 | 61 | 89 | 14 | [M+H]^+^ | 552.02/58.00 |
| Vancomycin | 20 | Medium | 5500 | 450 | 20 | 20 | 50 | 20 | 8 | [M+H]^+^ | 725.80/144.2 |
| Doxycycline | 20 | Medium | 5500 | 650 | 55 | 60 | 120 | 35 | 10 | [M+H]^+^ | 445.20/428.20 |

CUR = curtain gas; CAD = collision gas; IS = ion spray voltage; GS1 = ion source gas 1; GS2 = ion source gas 2, DP = declustering potential; CE = collision energy; CXP = collision exit potential; MRM = multiple reaction monitoring.

**Supplementary Figure 1**. Evaluation of potential tape interference during spatial drug quantitation. (a) The mimetic tissue was prepared by spiking 10 mg/g of each study drug and drug metabolite in lung homogenates, sequentially pouring into a cylindrical mold (a modified 2-mL syringe) followed by freezing, to assemble a plug containing 7 superposed cylinders of spiked tissue mimetic including blank control ^1^. Lung tissue was used because it adheres to adhesive film and tape more readily than hard bone tissue, which is essential to fully evaluate possible interference that may arise when sectioning onto such substrates. (b) The plug was removed from the mold by gently pushing the barrel of the syringe and cryosectioned at 10 μm. Serial sections were placed on either PET membrane or adhesive tape prior to LCM as illustrated.


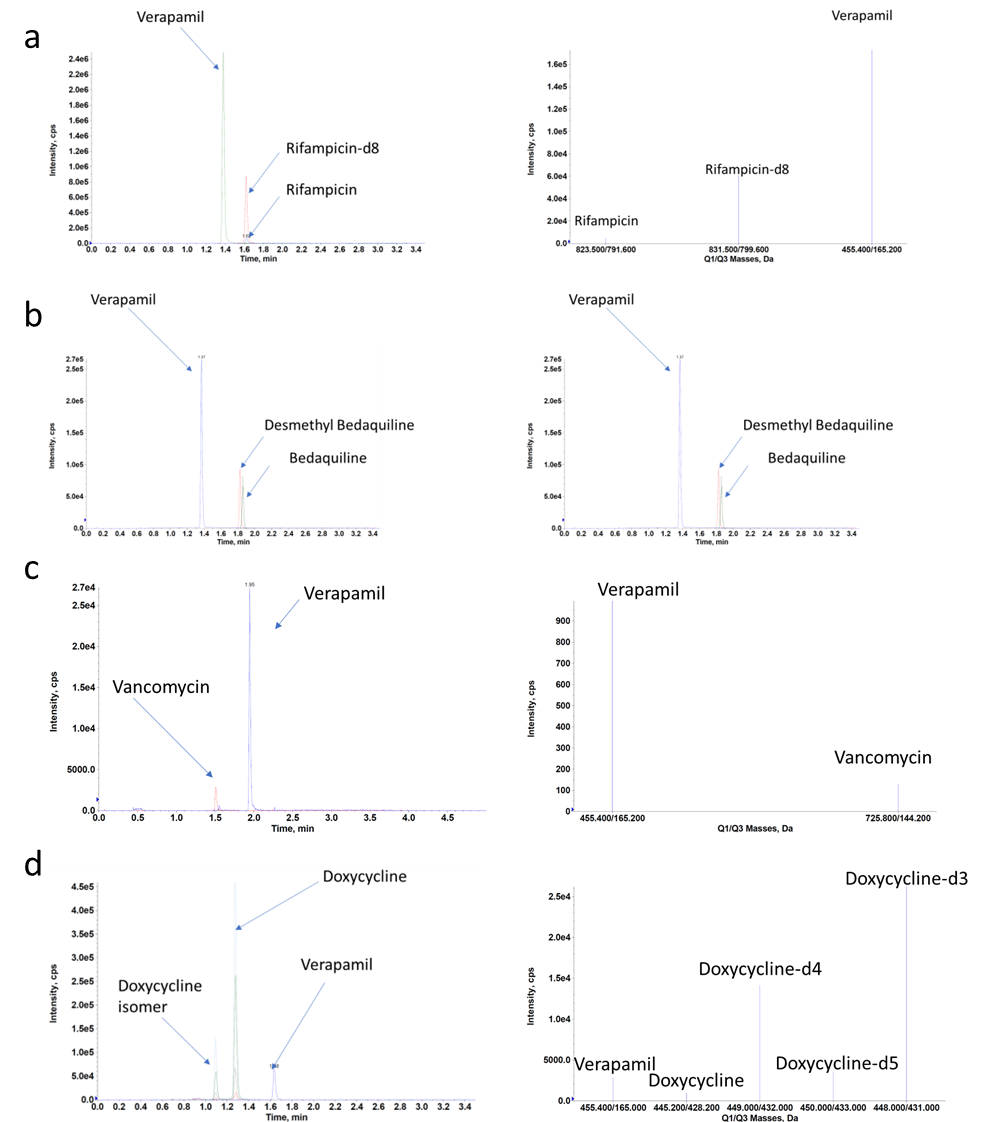
**Supplementary Figure 2.** Extracted ion Chromatograms (left panels) and MRM spectra (right panels) of rifampicin (a), bedaquiline (b), vancomycin (c), and doxycycline (d).

**Supplementary Figure 3**. LCM sample extraction time course. Samples dissected from bedaquiline containing tissues were extracted in 50/50 acetonitrile/methanol and sonicated as indicated for up to 15 min. The 60 min and 24 h samples were sonicated for 15 min and incubated in solvent for 45 min or overnight, respectively. They were sonicated again for 5 min immediately before analysis. (a) Peak area ration as a function of duration of extraction in organic solvent. Complete extraction was achieved within 5 min from all three bone tissue compartments. (b) % change in extraction efficiency centered on 10 min of sonication, the duration used in all experiments based on data shown in (a).

**Supplementary Figure 4**. Concentration time profile of bedaquiline and active metabolite desmethyl bedaquiline in rabbit plasma after 3 oral daily doses of 160 mg/kg, showing the slow absorption and narrow peak-to-trough window. Bones were collected from 3 rabbits 120h after the third and last drug dose.

**Supplementary Figure 5**. Chemical structure of bedaquiline and desmethyl-bedaquiline

REFERENCES

(1) Barry, J. A.; Groseclose, M. R.; Castellino, S. Quantification and assessment of detection capability in imaging mass spectrometry using a revised mimetic tissue model. *Bioanalysis* **2019**, *11* (11), 1099-1116. DOI: 10.4155/bio-2019-0035.

**Supplementary Movie 1**: Bone cryosectioning on adhesive film

**Supplementary Movie 2**: On-tape histology staining of bone section post MALDI MSI
